# How did COVID-19 affect medical and cardiology journals? A pandemic in literature

**DOI:** 10.2459/JCM.0000000000001245

**Published:** 2021-09-03

**Authors:** Andrea Venturelli, Marco Vitolo, Alessandro Albini, Giuseppe Boriani

**Affiliations:** a Cardiology Division, Department of Biomedical, Metabolic and Neural Sciences, University of Modena and Reggio Emilia, Policlinico di Modena; b Clinical and Experimental Medicine PhD Program, University of Modena and Reggio Emilia, Modena, Italy

**Keywords:** coronavirus, COVID-19, literature, medical journals, pandemic, SARS-CoV2

## Abstract

**Background and aims:**

The spreading speed of the COVID-19 pandemic forced the medical community to produce efforts in updating and sharing the evidence about this new disease, trying to preserve the accuracy of the data but at the same time avoiding the potentially harmful delay from discovery to implementation. The aim of our analysis was to assess the impact of the COVID-19 pandemic on medical literature in terms of proportion of COVID-19-related published papers and temporal patterns of publications within a sample of general/internal medicine and cardiology journals.

**Methods:**

We searched through PubMed scientific papers published from 1 January 2020 to 31 January 2021 about COVID-19 in ten major medical journals, of which five were in general/internal medicine and five in the cardiology field. We analyzed the proportion of COVID-19-related papers, and we examined temporal trends in the number of published papers.

**Results:**

Overall, the proportion of COVID-19-related papers was 18.5% (1986/10 756). This proportion was higher among the five selected general/internal medicine journals, compared with cardiology journals (23.8% vs 9.5%). The vast majority of papers were not original articles; in particular, in cardiology journals, there were 28% ‘original articles’, 17% ‘review articles’ and 55.1% ‘miscellaneous’, compared with 20.2%, 5.1% and 74.7% in general/internal medicine journals, respectively.

**Conclusions:**

Our analysis highlights the big impact of the COVID-19 pandemic on international scientific literature. General and internal medicine journals were mainly involved, with cardiology journals only at a later time.

## Introduction

In December 2019, a growing number of reports about a pneumonia cluster of unknown origin from Wuhan, China appeared in medical literature.^1^ Soon, a novel coronavirus was identified as the etiologic agent of these unusual cases.^2^ From that time, the ongoing coronavirus disease 19 (COVID-19) pandemic has caused more than 144 million confirmed cases of COVID-19, and more than 3 million deaths worldwide, at the time of 23 April 2021.^3^ The spreading speed of the COVID-19 pandemic forced the medical community to produce efforts in updating and sharing new evidence about this disease, trying to preserve the accuracy of the data but at the same time avoiding the potentially harmful delay from discovery to implementation.^4–6^ Scientists and healthcare workers had to face this ‘pandemic in literature’ to always keep up-to-date. At the same time, scientific evidence rapidly became the main tool to guide political, social and economic decisions that directly involved billions of people worldwide. For these reasons, the median time to publication of COVID-19-related articles has been shown to be much shorter compared with non-COVID-19-related articles.^7^

COVID-19 primarily involved emergency medicine, pulmonology and intensive care specialists, but soon cardiology departments too had to face the direct effect of the new coronavirus on the cardiovascular system and the impact of the pandemic on daily clinical activity.^8–12^

The aim of our analysis was to assess the impact of the COVID-19 pandemic on medical literature in terms of the proportion of COVID-19-related published papers and temporal patterns of publication within a sample of general/internal medicine and cardiology journals.

## Methods

### Search strategy and studies selection

We conducted a review of scientific papers published from 1 January 2020 to 31 January 2021 about COVID-19 in ten major medical journals. We selected the journals based on their impact factor, choosing between those with top-ranking positions, both in the field of medicine and in the field of cardiology. Also, since Italy was the first country involved in the pandemic outside of China, we selected two journals with an important Italian tradition and a series of contributions on COVID-19: one in the medical field, and one in the cardiology field, respectively. We selected the following journals: The New England Journal of Medicine (NEJM), The Lancet, Journal of the American Medical Association (JAMA), American Journal of Medicine (AJM), European Journal of Internal Medicine (EJIM) for general and internal medicine; Circulation, European Heart Journal (EHJ), Journal of the American College of Cardiology (JACC), Heart and Journal of Cardiovascular Medicine (JCM) for cardiology.

First, we calculated the total number of publications in the aforementioned journals during the analyzed period (from 1 January 2020 to 31 January 2021).

Second, we performed a selective literature search through PubMed using the following keywords: ‘SARS-CoV 2’, ‘COVID’, ‘COVID-19’ and their related terms in the same journals and for the same period.

Two investigators (A.V. and A.A.) independently screened records for eligibility based on title and abstracts. Two senior reviewers (M.V. and G.B.) independently analyzed the study selection and the data extraction process.

### Data analysis

We calculated the proportion of COVID-19-related papers in the total number of scientific papers published. Screened articles on COVID-19 were categorized as follows: ‘original article’ (including research letter and meta-analysis), ‘review article’ (including narrative and systematic review, position paper, consensus statements, guidelines) and ‘miscellaneous articles’ (including commentaries/editorials, opinion papers, viewpoints, perspectives, case reports, letters and replies). Corrigendum and retracted articles were excluded. Thereafter, selected papers were stratified according to publication date, month by month over the prespecified period consisting of 13 months.

Finally, since Italy was the first country affected by the COVID-19 pandemic outside of China, we performed a specific sub-analysis focusing on two journals that traditionally have an important number of contributions from Italy, i.e EJIM for general/internal medicine and JCM for cardiology.

## Results

**Fig. 1:**
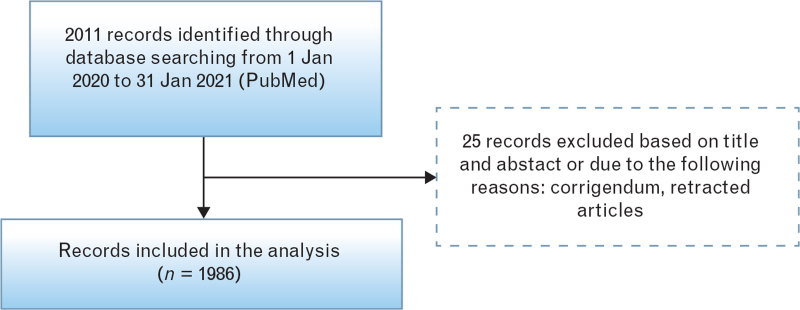
Flow diagram of the selection process of COVID-19-related papers.

A total number of 10 756 papers were published in the ten selected journals during the study period. Overall, 1986 papers regarding COVID-19 (18.5%) were identified after the exclusion of not pertinent, retracted or corrigendum articles (Fig. 1).

### Proportion of COVID-19-related articles

Among the five selected general/internal medicine journals 1602 papers were COVID-19-related over a total number of 6732 published papers (23.8%). In the five selected cardiology journals, the proportion of COVID-19-related papers was 9.5% (384/4024). Some differences among the selected journals were found in the proportion of COVID-19-related papers (Table 1).

**Table 1:** Total number of papers published from 1 January 2020 to 31 January 2021, with regard to all the fields covered by the specific journals, papers related to COVID-19 and proportion of COVID-19 in all published papers in the selected period

Journal name	Total papers	COVID-19-related	COVID-19-related/Total
General/internal medicine			
The New England Journal of Medicine	1882	472	25.1%
The Lancet	1705	556	32.6%
Journal of the American Medical Association	1836	457	24.9%
The American Journal of Medicine	834	47	5.6%
European Journal of Internal Medicine	475	70	14.7%
Cardiology			
Circulation	950	104	10.9%
European Heart Journal	1256	125	10.0%
Journal of the American College of Cardiology	1002	91	9.1%
Heart	591	40	6.8%
Journal of Cardiovascular Medicine	225	24	10.7%

### Temporal trends

**Fig. 2:**
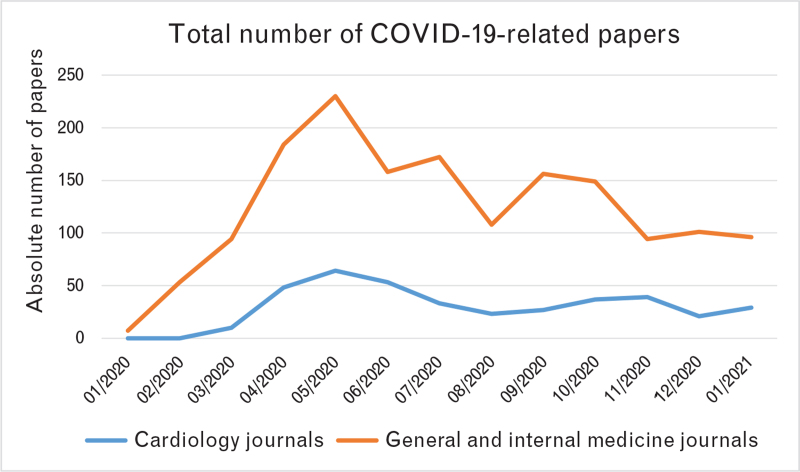
Side-by-side comparison of total number of COVID-19-related papers in general and internal medicine and cardiology journals.

**Fig. 3:**
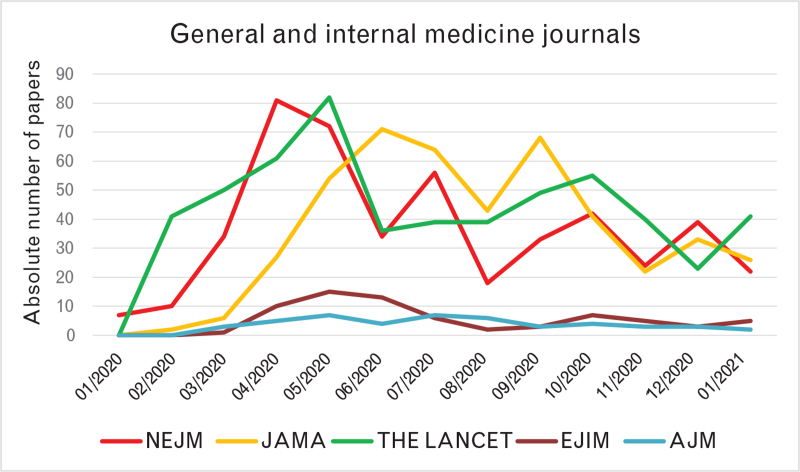
Temporal trend in COVID-19-related papers in general and internal medicine journals. AJM, American Journal of Medicine; EJIM, European Journal of Internal Medicine; JAMA, Journal of the American Medical Association; NEJM, The New England Journal of Medicine.

**Fig. 4:**
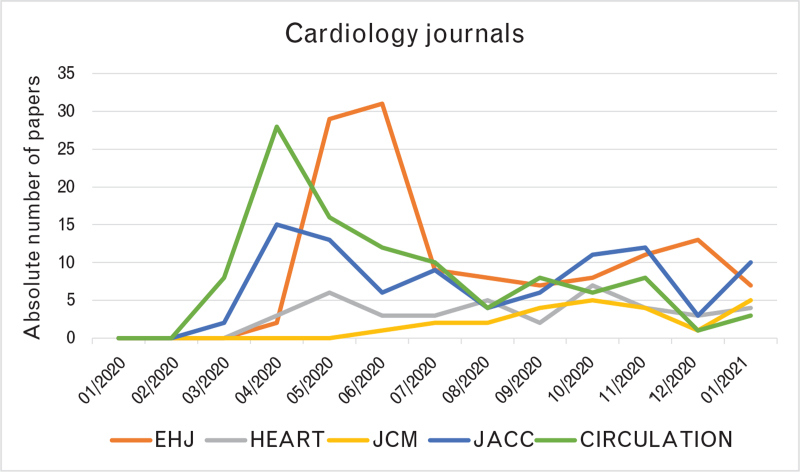
Temporal trend in COVID-19-related papers in cardiology journals. EHJ, European Heart Journal; JACC, Journal of the American College of Cardiology; JCM, Journal of Cardiovascular Medicine.

The analysis of publication temporal trends showed an exponential rise in the first 6 months of 2020, starting from the end of January 2020, with some delay of around 2–3 months in cardiology journals compared with general/internal medicine journals (Fig. 2). Indeed, the first COVID-19-related paper appeared in NEJM as Epub online on 24 January 2020,^2^ whereas the first in the cardiology journals group appeared in Circulation as Epub online on 17 March 2020.^8^ Publication trends in general/internal medicine journals revealed a smooth curve across the entire year 2020, without a real separation between the first and the second wave of the pandemic (Fig. 3). Conversely, in cardiology journals, we found a peak between April and June 2020, followed by a fall and a plateau phase with only a mild rise during the second wave of the pandemic in the late autumn (Fig. 4).

### Subgroup analysis

**Fig. 5:**
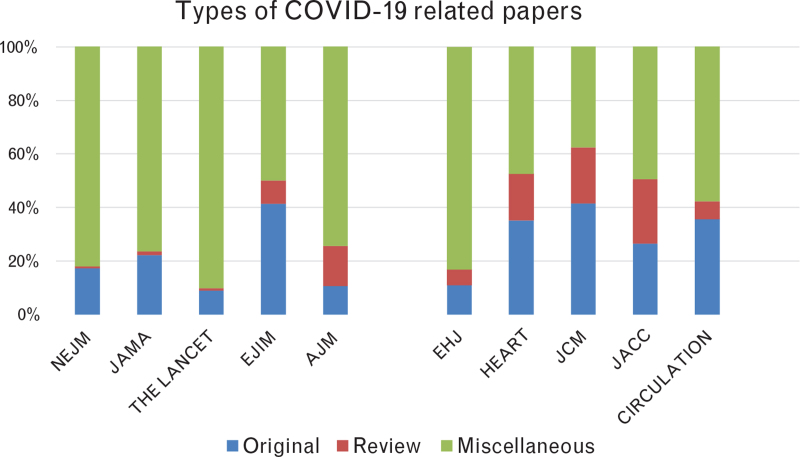
Different types of COVID-19-related papers among the selected journals: ‘original papers’, ‘review papers’ and ‘miscellaneous papers’ (i.e. commentaries/editorials, opinion papers, viewpoints, perspectives, case reports, letters and replies). AJM, American Journal of Medicine; EHJ, European Heart Journal; EJIM, European Journal of Internal Medicine; JACC, Journal of the American College of Cardiology; JAMA, Journal of the American Medical Association; JCM, Journal of Cardiovascular Medicine; NEJM, The New England Journal of Medicine.

In our analysis, among the total number of 1986 publications, we identified 367 ‘original articles’ (18%), 72 ‘review articles’ (4%) and 1547 papers in the ‘miscellaneous’ group (78%). On average, in cardiology journals, there were 99 ‘original articles’ (25.8%), 48 ‘review articles’ (12.5%) and 237 ‘miscellaneous’ (61.7%), compared with 268 (16.7%), 24 (1.5%) and 1310 (81.8%) in general/internal medicine journals, respectively (Fig. 5).

In the sub-analysis specifically focused on the journals with an important number of Italian contributions, we found that COVID-19-related papers from Italy (defined as first and/or the corresponding author from Italy) accounted for 71% (50/70) and 100% (24/24) in the EJIM and JCM, respectively. The original contributions published on EJIM^6,13–40^ (29/70 = 41.4%) and JCM^41–50^ (10/24 = 41.6%) constituted a substantial number of the COVID-19-related papers published in these journals: higher than what was published in the other analyzed journals (Fig. 5).

## Discussion

In our analysis, we focused on the impact of COVID-19 on medical literature specifically on general/internal medicine and cardiology journals. As expected, the main finding of our analysis was that COVID-19 strongly attracted the attention of the medical community across the entire year 2020.

A substantial amount of the scientific literature of the year 2020 was actually dedicated to COVID-19, although with some differences in temporal trends and number of published papers between journals dedicated to internal medicine or cardiology. This finding seems reasonably related to the fact that COVID-19 primarily involved emergency physicians, pulmonologists and intensivists and only in a subsequent period did the growing evidence of direct involvement of the cardiovascular system force the cardiology scientific community to focus on this disease. Indeed, this is well reflected by the delay in publication in cardiology journals compared with general and internal medicine journals.

The majority of papers regarding COVID-19 in our analysis were not reporting the results of clinical studies. The great number of commentaries, opinion papers and other ‘narrative’ types of articles was probably due to the initial lack of data about effective treatments, and due to the extraordinary impact not only on the healthcare system but also on social and economic factors. On average, the relative proportion of original articles is higher in cardiology journals, as compared with general and internal medicine journals where a large proportion of contributions were reviews, commentaries/editorials, opinion papers etc. Interestingly, original articles constitute a relevant part of the total published papers in the two journals of Italian tradition that we analyzed in our sub-analysis (JCM, EJIM), with the highest proportion among all the selected journals (41.7% in JCM and 41.4% in EJIM).

This high proportion in original articles among cardiology journals can be also the effect of the urgent need to obtain data to manage the complex setting regarding both patients with COVID-19 developing cardiovascular complications and patients with cardiovascular diseases being infected by the coronavirus and developing COVID-19. Some of these publications analyzed the implications of the COVID-19 pandemic on the organization of care in the community and reported a reduction in ST-segment elevation myocardial infarction (STEMI) admissions during the pandemic,^44^ as well as the impact of the pandemic on interventional activities for both coronary and electrophysiological procedures.^46,50^

Similarly, a reduction in the number of heart failure hospitalizations has also been observed, with a consequent worsening of prognosis.^51–54^ Possible explications may be attributable to the patient's fear of going to hospital with a delayed time to admission and therefore to a more advanced heart failure status. Also, modifications of hospital services and resources towards COVID-19 treatments could have left patients without usual care for their specific diseases. These analyses highlight how a pandemic can affect the severity, morbidity and mortality of patients without COVID-19 but with a serious medical condition, like heart failure.

At the same time, among patients with an implantable cardioverter defibrillator (ICD), a reduction in ventricular arrhythmias and ICD interventions during the period of the pandemic compared with the same period of the previous year was reported.^9,55^ Indeed, a reduction in the frequency of some manifestations of cardiovascular disease was observed during COVID-19, such as acute myocardial infarction^56^ or heart failure,^57^ but on the other hand an increase in out-of-hospital cardiac arrests, associated with reduced survival was observed.^58^ These findings further underline how complex the interaction is between different factors involving health of the population during COVID-19. Environmental factors, such as air pollution; social factors, such as lockdown, working and job changes; and emotional factors all acted in combination producing different effects that will be better understood only with longitudinal studies.

We also observed that, among the included papers regarding COVID-19, the most covered issues changed over time. At the beginning of the pandemic, the most frequent topics were about the disease, its clinical features and its prognostic and therapeutic implications.^12,59–63^ Subsequently, the scientific research focused on the pathophysiology and molecular mechanism,^64,65^ and finally the interest of literature moved towards epidemiological and social implications, with a specific interest also in the impact of COVID-19 in healthcare organization and scientific research.^66–71^ Interestingly, this pattern of the issues of papers fits the evolution over time of a pandemic; first the focus on the disease itself with the effort to limit the diffusion and the mortality, then the insights about molecular mechanisms and the development of therapeutic options, and finally the effect of the pandemic on the different aspects of society and the scientific world.

It has been reported how the COVID-19 pandemic led to an unprecedented rise in the production of scientific papers, with a tendency to accelerate the process of publication as shown by the high number of scientific contributions presented as preprint before peer review.^72^ The low rate of publications for contributions presented as preprint (<6%) raises concern about the scientific value of the same contributions proposed during the COVID-19 pandemic and this phenomenon surely requires some form of regulation in the future. Also, the issue of article withdrawals from some top-ranked journals^73,74^ suggests that the traditional process of peer review may have been challenged by the pressures exerted by a disruptive phenomenon such as the COVID-19 pandemic. The same pressure acted on the use of social media, widely used for disseminating scientific information during the pandemic.^75^ It appears that the pandemic markedly affected the process of the evaluation and presentation of scientific contributions^76^ and this had a variable impact on journals in the field of general and internal medicine, whose future dynamics need to be carefully monitored.

The scientific community therefore must be aware of the risks related to the need for rapid publishing and should always pursue the good practices of the editorial process. This issue of maintaining a high-quality standard of scientific publications during the pandemic has raised particular interest. As reported, our analysis shows that in the fields of both cardiology and general/internal medicine, original papers were only a fraction of the total number of publications in these fields, since a large number of the publications during the year 2020 were actually editorials, reviews or commentaries. This finding extends a previous observation limited to the first 4 months of year 2020.^77^

During the pandemic, even more than at other times, the risk of misleading or confounding information due to the need for rapid data sharing could be relevant. As a matter of fact, we found a not negligible number of retracted and corrigendum articles (seven and nine, respectively). Some of these articles dealt directly with cardiologic treatments in patients with COVID-19, e.g. the use of angiotensin-converting enzyme inhibitors and angiotensin II receptor blockers.^78^ Other examples are studies about the use and efficacy of hydroxychloroquine or chloroquine, or tobacco smoking and the severity of COVID-19.^79,80^

In this situation, the scientific community should be targeted to provide reliable information following the best contributions of scientists through evidence-based medicine.

### Limitations

This study is based on the analysis of a sample of ten medical journals that are of course not fully representative of all scientific literature. We also selected these journals arbitrarily based on their reputation and importance in their field, as well as based on their Italian tradition and contributions, specifically for EJIM and JCM. Also, the period of time considered is limited to 13 months, excluding the first months of 2021. Another limitation is linked to the categorization of papers: we put together different types of articles in the ‘review’ and ‘miscellaneous’ groups, favouring a simple and clear way to display the results instead of their fragmentation in many sub-types.

## Conclusion

Our analysis showed how international scientific literature has been significantly involved in the COVID-19 pandemic. Particularly, a relevant impact occurred in general and internal medicine journals, but original studies were proportionally more prevalent in cardiology journals. With such an explosion of articles of different nature, aim and type, and being that the vast majority of publications are not original articles, it appears that COVID-19 induced a pandemic also in the medical literature.

## Conflicts of interest

The authors report no conflicts of interest related to the present article. G.B. reported small speaker fees from Medtronic, Boston, Boehringer Ingelheim and Bayer outside of the submitted work.
